# Lily NIN-like transcription factor LlNLP7 confers thermotolerance and promotes growth

**DOI:** 10.1186/s43897-026-00253-8

**Published:** 2026-08-10

**Authors:** Ziwei Liao, Jun Xiang, Yinyi Zhang, Xue Gong, Ze Wu, Nianjun Teng

**Affiliations:** 1 Key Laboratory of Flower Biology and Germplasm Innovation, Key Laboratory of Landscaping, Ministry of Agriculture and Rural Affairs, College of Horticulture, Nanjing Agricultural University, Nanjing, 211800 China; 2 Nanjing Agricultural University-Nanjing Oriole Island Modern Agricultural Development Co. Ltd., Lily Science and Technology Backyard Qixia of Jiangsu/Jiangsu Graduate Workstation, Nanjing, 210043 China

In *Arabidopsis*, NLP7 (NIN-LIKE PROTEIN 7) has been established as a nitrate (NO_3_^−^) receptor and a key regulator in plant nitrogen signaling pathways, playing an indispensable role in nitrogen absorption and utilization (Konishi and Yanagisawa 2013; Liu et al. 2022). Additionally, under cold stress, NLP7 is phosphorylated by CPK28 (CALCIUM-DEPENDENT PROTEIN KINASE 28), directly activating the expression of downstream cold-responsive genes (Ding et al. 2022). Recently, NLP7 homologs of rice and *Arabidopsis* have been reported to play roles in thermotolerance (Zhao et al. 2025; Zhu et al. 2025). However, research on the involvement of NLP7 in temperature stress responses in horticultural crops are scarce.

Lily (*Lilium* spp.), an important floral crop, exhibits poor thermotolerance, with high temperatures severely affecting its quality and significantly limiting year-round production (Li et al. 2024; Wu et al. 2026). HSFs (HEAT STRESS TRANSCRIPTION FACTORs) are central regulatory components in the plant response to high temperatures; as terminal components in the heat signaling pathway, they directly regulate heat-responsive genes for thermotolerance establishment (Kan et al. 2023). LlHSFA3A is a critical HSF member mediating thermotolerance in lily (Wu et al. 2018, 2019, 2024), yet its upstream regulatory mechanisms are not well understood. Through a yeast one-hybrid (Y1H) library screening, we identified LlNLP7 as a potential direct regulator of *LlHSFA3A*. Phylogenetic analysis with NIN-like transcription factor family of *Arabidopsi*s positioned LlNLP7 closely to AtNLP7, thus naming it LlNLP7 (Fig. S1). Protein alignment analysis of NLP7 homologs identified the typical RWP-RK and PB1 domains of NIN-like transcription factors at the C-terminus of LlNLP7 (Fig. S1). To investigate the activation of *LlNLP7* by high temperatures, we conducted real-time quantitative PCR (RT-qPCR) analysis on lily leaves exposed to heat stress. The RT-qPCR result indicated that the expression of *LlNLP7* was rapidly activated after 0.5 h of heat treatment, sustaining expression up to 12 h, suggesting a prolonged activation by heat stress (Fig. 1A). The transient expression of a GFP fusion protein in tobacco leaf cells revealed that the GFP fluorescence of GFP-LlNLP7 partially overlapped with a nuclear marker (NLS-RFP), indicating that LlNLP7 was partially localized in the nucleus under normal conditions and belonged to a nuclear-cytoplasmic localization protein; however, under heat stress conditions, GFP-LlNLP7 predominantly localized in the nucleus, suggesting that high temperatures promoted the nuclear translocation of LlNLP7 (Fig. 1B, S2). The result of nucleus-cytoplasm fractionation experiment also confirmed this observation (Fig. S2). In addition, we established stable overexpression of *GFP-LlNLP7* in lily by transforming embryonic calli (Xiang et al. 2025) (Fig. S3). Similarly, it was also observed that the fluorescent signal in *GFP-LlNLP7* transgenic lily root cells translocated from the cytoplasm to the nucleus under heat stress (Fig. 1C, S4).

**Fig.1:**
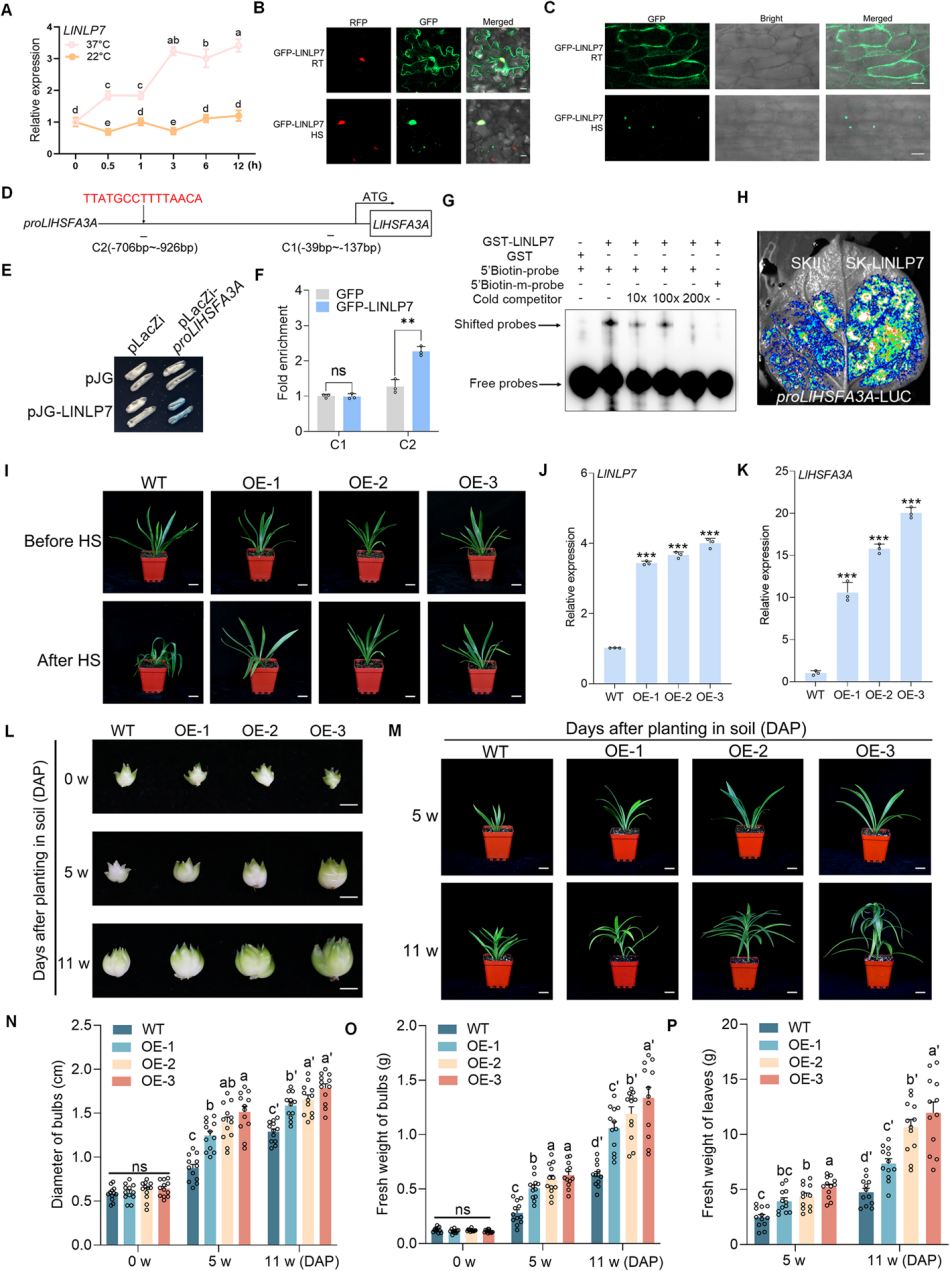
Overexpression of *LlNLP7* in lily confers thermotolerance and promotes growth. **A** Expression assay of *LlNLP7* in lily leaves under normal (22 °C) and heat stress (HS, 37℃) for different durations. Data are presented as the mean ± SD from three replicates, with different letters indicating statistically significant difference (Student–Newman–Keuls test, *P* < 0.05). **B** Subcellular localization of LlNLP7 in tobacco leaves. Confocal microscopy images showing the localization of GFP-LlNLP7 in tobacco (*Nicotiana benthamiana*) leaf epidermal cells co-expressing the nuclear marker RFP-NLS under control (22 °C) or heat stress (37 °C, 30 min) conditions. The merged channels show co-localization of GFP and RFP signals. Scale bar = 100 µm. **C** Subcellular localization of LlNLP7 in transgenic lily root cells. Confocal microscopy images showing the localization of GFP-LlNLP7 in lily root cells under control (22 °C) or heat stress (37 °C, 30 min) conditions. Scale bar = 100 µm. **D** Diagram of the *LlHSFA3A* promoter. The potential nitrate-responsive *cis*-element (NRE) is marked with an arrow. The C1 and C2 fragments used for ChIP-qPCR assay are indicated with black lines. The C2 fragment contains a potential NRE, while the C1 fragment serves as a negative control, lacking this element. **E** Y1H assay. Yeast cells were co-transformed with the LlNLP7 effector and the *pLlHSFA3A*-*LacZ* reporter, and grown on SD/-Trp/-Ura medium supplemented with X-Gal. A representative image from three independent replicates is shown. **F** ChIP-qPCR assay. Enrichment of the *LlHSFA3A* promoter fragment bound by GFP-LlNLP7 in transiently transformed lily calli, empty GFP transformants used as a control. The C1 fragment served as a negative control region. Data are mean ± SD of three replicates (Student’s *t*-test, **P < 0.01). ns, no significant difference. **G** EMSA. Purified GST-LlNLP7 protein was incubated with a labeled NRE probe derived from the *LlHSFA3A* promoter. The m-probe indicates the NRE mutant labeled probe. The unlabeled competitive probe was added in specific reactions. A representative image from three independent experiments is shown. **H** Detection of the Luc signal in tobacco leaves. Representative image based on three experiments. **I** Phenotypes of *LlNLP7*-overexpressing plants under heat stress. Wild-type (WT) and *LlNLP7*-overexpressing (OE) plants were exposed to heat stress (HS, 45 °C, 5 h) and allowed to recovery for 5 days at 22 °C. Scale bar = 2 cm. **J** Expression of *LlNLP7* in leaves of *LlNLP7*-overexpressing (OE) lily plants. Data are presented as the mean ± SD of three replicates (Student’s *t*-test, ***P < 0.001). **K** Expression of *LlHSFA3A* in leaves of *LlNLP7*-overexpressing (OE) lily plants. Data are presented as the mean ± SD of three replicates (Student’s *t*-test, ***P < 0.001).** L** Phenotypes of wild-type (WT) and *LlNLP7*-overexpressing (OE) lily bulbs at 0 weeks (before planting), 5 weeks, and 11 weeks after planting. Scale bar = 1 cm. **M** Growth phenotypes of wild-type (WT) and *LlNLP7*-overexpressing (OE) lily plants at different developmental stages. DAP, days after planting in soil. w, weeks. Scale bar = 2 cm. **N** Bulb diameter of wild-type (WT) and *LlNLP7*-overexpressing (OE) lily plants at the indicated time points. Data are means ± SD, with different letters indicating statistically significant difference (n = 12, Student–Newman–Keuls test, *P* < 0.05). ns, no significant difference. **O** Fresh weight of bulbs from wild-type (WT) and *LlNLP7*-overexpressing (OE) lily plants. Data are means ± SD, with different letters indicating statistically significant difference (n = 12, Student–Newman–Keuls test, *P* < 0.05). ns, no significant difference. **P** Fresh weight of the leaves of wild-type (WT) and *LlNLP*7-overexpressing (OE) lily plants. Data are means ± SD, with different letters indicating statistically significant difference (n = 12, Student–Newman–Keuls test, *P* < 0.05). ns, no significant difference. DAP, days after planting in soil. w, weeks

Subsequently, we assessed the transcriptional activity of LlNLP7 using a yeast system. The results demonstrated that yeast cells expressing the full-length LlNLP7 grew well on nutrient-deficient SD medium, indicating that LlNLP7 exhibited transactivation activity in yeast cells (Fig. S5). Protein truncation analysis of LlNLP7 revealed that its N-terminus possessed transcriptional activation capability, with the activation domain located in the N-terminal P5 region (Fig. S5). Next, we constructed the pEAQ-LlNLP7, pEAQ-LlNLP7-P5, and pEAQ-LlNLP7-P9 vectors and employed a dual-luciferase reporter system in tobacco leaves to assess their transcriptional ability in plant cells. The results showed that, compared to the expression of the BD protein alone, both BD-LlNLP7 and BD-LlNLP7-P5 significantly activated the expression of the *GAL4 UAS* -driven LUC (luciferase) reporter, but the BD-LlNLP7-P9 did not (Fig. S5). These data indicate that LlNLP7 also exhibits transactivation capability in plant cells, and its N-terminal P5 region contributes the transactivation ability.

A Y1H assay confirmed that LlNLP7 directly bound to the promoter of *LlHSFA3A* (Fig. 1D,E). ChIP-qPCR (Chromatin immunoprecipitation quantitative PCR) analysis indicated that LlNLP7 associated with a region containing nitrate-responsive *cis*-element (NRE, 5’-TG[A/G]C[C/T]CTT-3’) within the *LlHSFA3A* promoter in vivo (Fig. 1F). In addition, EMSA (electrophoretic mobility shift assay) demonstrated that LlNLP7 bound to the biotin-labeled probes containing NRE in vitro, while it did not bind to a mutated probe (Fig. 1G). Using a tobacco transient expression system, we performed a dual-luciferase reporter assay to investigate the binding effect of LlNLP7 to the *LlHSFA3A* promoter. The results showed that, compared to the control, the transient overexpression of *LlNLP7* in tobacco leaves enhanced the LUC activity driven by the *LlHSFA3A* promoter (Fig. 1H, S6), indicating that LlNLP7 activates the expression of *LlHSFA3A* by directly binding to its promoter.

To investigate the role of LlNLP7 in lily, we detected the thermotolerance of *LlNLP7*-overexpressing lines (Fig. 1I). Gene expression analysis revealed an enhanced expression of *LlNLP7* in the transgenic lines, along with a significant increase in the expression of *LlHSFA3A* (Fig. 1J,K). Except that, we observed that *LlHSFA1* and *LlHSFA2* were also activated by *LlNLP7* overexpression (Fig. S7). Wild-type and overexpressing lily plants with consistent growth were selected as experimental materials (Fig. 1I). After heat stress treatment, the overexpressing plants exhibited less leaf wilting and chlorosis compared to the wild-type plants, with lower relative ion leakage and higher chlorophyll content (Fig. S7). DAB and NBT staining assays indicated that the overexpressing plants accumulated less ROS under heat stress (Fig. S7). The detections of H_2_O_2_ and •O₂⁻ contents further showed the similar results (Fig. S7). These data suggest that overexpression of *LlNLP7* alleviates heat-induced cellular damage and enhances resistance to heat stress in lily. Additionally, we utilized a Tobacco Rattle Virus (TRV)—mediated Virus-Induced Gene Silencing (VIGS) system to silence *LlNLP7* expression in lily plants, followed by heat stress treatment to evaluate their thermotolerance (Fig. S8). In lily plants with silenced *LlNLP7*, the expression of *LlHSFA3A* was suppressed at both normal temperature and high temperature conditions, as well *LlHSFA1* and *LlHSFA2* (Fig. S8), indicating that LlNLP7 was essential for their heat-induced expression. Following heat stress treatment, *LlNLP7*-silenced lily plants displayed greater leaf wilting and chlorosis compared to the TRV-control plants (Fig. S8). Under heat stress conditions, *LlNLP7*-silenced plants exhibited more severe damage, higher relative ion leakage, decreased chlorophyll accumulation, and an increase in ROS levels (Fig. S8). These results demonstrated that silencing *LlNLP7* compromised the ability of lily cells to withstand high-temperature environments. Overall, these data indicate that LlNLP7 plays a positive role in thermotolerance.

NLP7 has been implicated as a central hub for primary nitrate responses, with CPK10, CPK30, and CPK32 promoting nuclear retention of NLP7 through phosphorylation upon nitrate response (Konishi and Yanagisawa 2013; Liu et al. 2017). CPK28 interacts with NLP7 in a calcium-dependent manner, enhancing its role in cold stress responses (Ding et al. 2022). Recent research in *Arabidopsis* has indicated that the heat-triggered translocation of NLP7 is independent of nitrate (Zhao et al. 2025); similarly, in rice, the NLP7 homology OsNLP3 also translocates to the nucleus in response to heat stress (Zhu et al. 2025); however, whether they also participate in heat stress responses through phosphorylation modifications remains to be further investigated. Our findings demonstrate that lily *LlNLP7* is induced by high temperatures, with the protein translocating to the nucleus under heat stress to activate the expression of *LlHSFA3A*, thereby contributing to the establishment of thermotolerance. Although our results demonstrated that LlNLP7 directly activates the expression of *LlHSFA3A*, we also observed distinct expression patterns between *LlNLP7* and *LlHSFA3A*. *LlNLP7* was continuously induced by high temperatures, while the expression of *LlHSFA3A* exhibited a decline in heat-inducible expression following prolonged heat stress (Wu et al. 2018). These seemingly contradictory findings suggest that the expression of *LlHSFA3A* may be regulated by additional factors, such as the activation of potential transcriptional repressors under extended heat stress conditions, which could inhibit *LlHSFA3A* expression. Furthermore, in the context of nitrate response, there exists a negative feedback loop within the NLP7-mediated regulatory pathway, wherein the accumulation of NLP7 directly activates downstream repressors that suppress its own function (Ruffel et al. 2025). Whether a similar feedback mechanism operates in response to heat stress remains to be elucidated. Nevertheless, recent studies have found a similar NLP-HSF regulatory module in *Arabidopsis* and rice (Zhao et al. 2025; Zhu et al. 2025), indicating that this module is conserved across different plant species. NLP7 is not only involved in nitrate uptake and assimilation but also plays a conserved role in the temperature stress response in plants.

Importantly, we also observed that, compared to wild-type plants, the *LlNLP7*-overexpressing lily lines exhibited faster growth, with a bigger bulb and increased plant height (Fig. 1L-P, S9). Similar results have been observed in *Arabidopsis*, where the overexpression of its own *NLP7*, as well as the rice *OsNLP3* or maize *ZmNLP6* and *ZmNLP8*, significantly improves growth (Yu et al. 2016; Cao et al. 2017; Zhang et al. 2022). These findings, along with our results, indicate that homologs of NLP7 play a similar role in promoting growth across different species, including dicotyledonous plants, monocot crops, and non-grass monocot horticultural plants. These results also strongly suggests that NLP7 ortholog is a potential breeding target gene with significant practical value. Overexpression of *NLP7* not only enhances plants’ tolerance to both low and high temperatures but also promotes growth. Such characteristics indicate that NLP7 has broad application prospects in crop improvement and breeding, providing new strategies for increasing agricultural productivity and addressing climate change. Its dual role in enhancing crop resilience and promoting growth holds promise for delivering significant benefits to future agricultural development.

## Supplementary Information

Supplementary Material 1.

Supplementary Material 2,

Supplementary Material 3,

## Data Availability

All data are available in the manuscript.
