# Supplementary material for: Lily NIN-like transcription factor LlNLP7 confers thermotolerance and promotes growth
Source: Mol Hortic. 2026 Aug 10;6:57. doi: 10.1186/s43897-026-00253-8 (PMC13455299; doi:10.1186/s43897-026-00253-8)
Supplement: Supplementary file 1 [file 43897_2026_253_MOESM1_ESM.docx]

**Figure S1. LlNLP7 is a member of the NLP family.** **(A)** Phylogenetic analysis of LlNLP7 and NLP family members from *Arabidopsis thaliana*. Bootstrap values from 1000 replicates are indicated at the nodes. The scale bar represents the number of amino acid substitutions per site. **(B)** Multiple sequence alignment of the amino acid sequences of NLP7 homologs. RWP-PK domain is marked with black line, and the Phox and Bem1 (PB1) domain is marked with black line. The species and protein accession numbers are as follows: LlNLP7 (*Lilium longiflorum*), CnNLP7 (*Cocos nucifera*, EHA8590108.1), EgNLP7 (*Elaeis guineensis*, XP_010912370.3), CmNLP7 (*Cucurbita moschata*, KAG7031068.1), PdNLP7 (*Phoenix dactylifera*, XP_008777047.2).

**Figure S2. Heat stress promotes the translocation of LlNLP7 into the nucleus. (A)** The fluorescence signals of LlNLP7-GFP in tobacco leaves under the control condition (22°C) and heat stress condition (37 °C, 30 min) were observed separately using a laser scanning confocal microscope, and the GFP fluorescence intensity was quantitatively analyzed by ImageJ software. Data are means ± SD (n = 40, Student’s *t*-test, ****P* < 0.001). **(B)** *GFP-LlNLP7* was transiently expressed in tobacco leaves, followed by a 48-h incubation period, after which the samples were subjected to heat treatment (37°C for 30 min). The accumulation of GFP-LlNLP7 proteins in the cytoplasm and nucleus was evaluated using a nuclear-cytoplasmic protein separation technique followed by Western blot (WB) analysis. Specific antibodies were employed to target the GFP (Green Fluorescent Protein), as well as the nuclear internal control (Histone 3, H3) and the cytoplasmic internal control (Actin) to ensure accurate labeling. Lanes 1 and 2 display the WB results for the cytoplasm under room temperature (RT, 22°C) and heat stress (HS, 37°C for 30 min) conditions, respectively, while Lanes 3 and 4 show the WB results for the nucleus under RT and HS conditions, respectively.

**Figure S3. Molecular and cellular characterization of *LlNLP7-*overexpressing lily lines. (A)** PCR identification of transgenic lily plants. Genomic DNA PCR was performed using gene-specific primers to amplify the GFP-LlNLP7 fusion fragment. CK, plasmid positive control; WT, wild-type plant (negative control). **(B)** Detection of GFP signal in root tips of *LlNLP7*-overexpressing (OE) lily plants. Scale bar = 100 µm.

**Figure S4. Heat stress promotes the translocation of LlNLP7 into the nucleus of the transgenic lily root cells.** The fluorescence signals of GFP-LlNLP7 in lily root cells under the control condition (22℃) and heat stress condition (37℃, 30 min) were observed separately using a laser scanning confocal microscope, and the GFP fluorescence intensity was quantitatively analyzed by ImageJ software. Data are means ± SD (n = 40, Student’s *t*-test, ****P* < 0.001).

**Figure S5. LlNLP7 exhibits transcriptional activation activity in yeast and plants. (A)** Schematic of full-length and truncated LlNLP7 proteins used in this study. **(B)** Yeast transactivation assay. Yeast strain AH109 cells expressing the indicated constructs were grown on synthetic defined (SD) medium without Trp (SD/-Trp) or without Trp and His (SD/-Trp/-His) supplemented with different concentrations of 3-AT (3-amino-1,2,4-triazole). BD and GAL4 serve as negative and positive controls, respectively. **(C)** Quantitative β-galactosidase activity assay of the yeast transformants in **(B)**. Data are mean ± SD of three replicates, with different letters indicating statistically significant difference (Student-Newman-Keuls test, *P* < 0.05). **(D)** The constructs used for the transactivation activity assay in tobacco leaves. **(E)** Transcriptional activation assay of full-length LlNLP7 in tobacco leaves. Scale bar = 1 cm. **(F)** Transcriptional activation assay of LlNLP7-P5 in tobacco leaves. Scale bar = 1 cm. **(G)** Transcriptional activation assay of LlNLP7-P9 in tobacco leaves. Scale bar = 1 cm. **(H)** Quantification of relative LUC intensity from **(E)**. Data are mean ± SD of three replicates (Student’s *t*-test, **P* < 0.001). **(I)** Quantification of relative LUC intensity from **(F)**. Data are mean ± SD of three replicates (Student’s *t*-test, ****P* < 0.001). **(J)** Quantification of relative LUC intensity from **(G)**. Data are mean ± SD of three replicates (Student’s *t*-test, ns, no significant difference).

**Figure S6. LlNLP7 binds to the *LlHSFA3A* promoter and enhances its activity. (A)** Schematic of the effector and reporter constructs for the dual-luciferase assay. The effector plasmid expresses *LlNLP7* driven by the *CaMV 35S* promoter. The reporter plasmid contains the firefly luciferase (LUC) gene fused to the *LlHSFA3A* promoter. **(B)** Relative LUC activity in tobacco leaves co-infiltrated with the constructs shown in **(A)**. Data are means ± SD of three replicates (Student’s *t*-test, ***P* < 0.01).

**Figure S7. Overexpression of *LlNLP7* enhances thermotolerance of lily. (A)** Expression of *LlHSFA1* in leaves of *LlNLP7*-overexpressing (OE) lily plants. Data are presented as the mean ± SD of three replicates (Student’s *t*-test, **P* < 0.05). **(B)** Expression of *LlHSFA2* in leaves of *LlNLP7*-overexpressing (OE) lily plants. Data are presented as the mean ± SD of three replicates (Student’s *t*-test, ***P* < 0.01). **(C)** Relative ion leakage in wild-type (WT) and *LlNLP7*-overexpressing (OE) lily under control (22°C) or heat stress (45°C, 5 h) conditions. Data are means ± SD of three replicates, with different letters indicating statistically significant difference (Student-Newman-Keuls test, *P* < 0.05). **(D)** Total chlorophyll content in wild-type (WT) and *LlNLP7*-overexpressing (OE) lily plants under control (22°C) or heat stress (45°C, 5 h) conditions. Data are means ± SD of three replicates, with different letters indicating statistically significant difference (Student-Newman-Keuls test, *P* < 0.05). **(E)** DAB staining detecting hydrogen peroxide (H₂O₂) accumulation in leaves of wild-type (WT) and *LlNLP7*-overexpressing (OE) lily plants before (RT, 22°C) and after heat stress (HS, 45°C, 5 h). Scale bar = 1 cm. **(F)** NBT staining detecting superoxide anion (·O_2_^-^) accumulation in leaves of wild-type (WT) and *LlNLP7*-overexpressing (OE) lily plants before (RT, 22°C) and after heat stress (HS, 45°C, 5 h). Scale bar = 1 cm. **(G)** H_2_O_2_ content in leaves of wild-type (WT) and *LlNLP7*-overexpressing (OE) lily plants under control (22°C) or heat stress (45°C, 5 h) conditions. Data are means ± SD of three replicates, with different letters indicating statistically significant difference (Student-Newman-Keuls test, *P* < 0.05). **(H)** ·O_2_^-^ content in leaves of wild-type (WT) and *LlNLP7*-overexpressing (OE) lily plants under control (22°C) or heat stress (45°C, 5 h) conditions. Data are means ± SD of three replicates, with different letters indicating statistically significant difference (Student-Newman-Keuls test, *P* < 0.05).

**Figure S8. Silencing of *LlNLP7* reduces thermotolerance of lily.** **(A)** Phenotypes of TRV-control and *LlNLP7-*silencing (TRV-LlNLP7) lily plants before and after heat stress (HS, 45°C, 5 h). Scale bar = 2 cm. **(B)** The expression of *LlNLP7* in TRV-control and *LlNLP7-*silencing (TRV-LlNLP7) lily plants under control (22°C) or heat stress (45°C, 1 h) conditions. Data are means ± SD of three replicates (Student’s *t*-test, ****P* < 0.001). **(C)** The expression of *LlHSFA1* in TRV-control and *LlNLP7-*silencing (TRV-LlNLP7) lily plants under control (22°C) or heat stress (45°C, 1 h) conditions. Data are means ± SD of three replicates (Student’s *t*-test, ***P* < 0.01). **(D)** The expression of *LlHSFA2* in TRV-control and *LlNLP7-*silencing (TRV-LlNLP7) lily plants under control (22°C) or heat stress (45°C, 1 h) conditions. Data are means ± SD of three replicates (Student’s *t*-test, ***P* < 0.01). **(E)** The expression of *LlHSFA3A* in TRV-control and *LlNLP7-*silencing (TRV-LlNLP7) lily plants under control (22°C) or heat stress (45°C, 1 h) conditions. Data are means ± SD of three replicates (Student’s *t*-test, ****P* < 0.001). **(F)** Relative ion leakage in TRV-control and *LlNLP7-*silencing (TRV-LlNLP7) lily plants under control (22°C) or heat stress (45°C, 5 h) conditions. Data are means ± SD of three replicates (Student’s *t*-test, ****P* < 0.001). **(G)** Total chlorophyll content in TRV-control and *LlNLP7*-silencing (TRV-LlNLP7) lily plants under control (22°C) or heat stress (45°C, 5 h) conditions. Data are means ± SD of three replicates (Student’s *t*-test, ****P* < 0.001). **(H)** DAB staining detecting hydrogen peroxide (H_2_O_2_) accumulation in leaves of TRV-control and *LlNLP7*-silencing (TRV-LlNLP7) lily plants before and after heat stress (45°C, 5 h). Scale bar = 1 cm. **(I)** NBT staining detecting superoxide anion (·O_2_^-^) accumulation in leaves of TRV-control and *LlNLP7*-silencing (TRV-LlNLP7) lily plants before and after heat stress (45°C, 5 h). Scale bar = 1 cm. **(J)** H_2_O_2_ content in leaves of TRV-control and *LlNLP7*-silencing (TRV-LlNLP7) lily plants under control (22°C) or heat stress (45°C, 5 h) conditions. Data are means ± SD of three replicates (Student’s *t*-test, ****P* < 0.001). **(K)** ·O_2_^-^ content in leaves of TRV-control and *LlNLP7*-silencing (TRV-LlNLP7) lily plants under control (22°C) or heat stress (45°C, 5 h) conditions. Data are means ± SD of three replicates (Student’s *t*-test, ****P* < 0.001).

**Figure S9. Overexpression of *LlNLP7* promotes growth in lily. (A)** Leaf numbers of per plant in wild-type (WT) and *LlNLP7*-overexpressing (OE) lines. Data are means ± SD (n = 12, Student-Newman-Keuls test, *P* < 0.05). **(B)** Plant height of wild-type (WT) and *LlNLP7*-overexpressing (OE) lines at 11 weeks after planting. Data are means ± SD (n = 12, Student-Newman-Keuls test, *P* < 0.05).
