# Supplementary material for: Lily NIN-like transcription factor LlNLP7 confers thermotolerance and promotes growth
Source: Mol Hortic. 2026 Aug 10;6:57. doi: 10.1186/s43897-026-00253-8 (PMC13455299; doi:10.1186/s43897-026-00253-8)
Supplement: Supplementary file 2 [file 43897_2026_253_MOESM2_ESM.docx]

**Table S1** Primers used for RT-qPCR.

| Gene name | Sequence |
| --- | --- |
| *18S rRNA* | 5’-AGTTGGTGGAGCGATTTGTCT-3’ |
|  | 5’-CCTGTTATTGCCTCAAACTTCC-3’ |
| *LlNLP7* | 5’-TCAGTGGAGACTGATATGCCTC-3’ |
|  | 5’-CTGCCACCAAACTTAGCCTCTTCG-3’ |
| *LlHSFA1* | 5’-ATGGGAAGTGTCTATGTGGGG-3’ |
|  | 5’-CATTGATACTTGGCAGTTGTTGG-3’ |
| *LlHSFA2* | 5’-CAGACTGAGGTCGAGTTGGAAG-3’ |
|  | 5’-AACACAGCCCTCTTATCTTCTCG-3’ |
| *LlHSFA3A* | 5’-CAGTTCACCTTATCCGCTGCGA-3’ |
|  | 5’-CTTAAAGTTCAGATGGTCGTGTCCTTG-3’ |

**Table S2** Primers used for plasmid reconstruction.

| Empty vector | Vector name | Sequence |
| --- | --- | --- |
| pCAMBIA1300- GFP | p1300-LlNLP7-GFP | 5’-CTCGGCATGGACGAGCTGTACAAGGTCGAC  ATGTCGAACCCCGGCGCCGACGATT-3 |
|  |  | 5’-ATGTTTGAACGATCGGGGAAATTCGAGCTC  TCAATCGACCGTGCTATCACATGAG-3’ |
| pGreenⅡ62-SK | SK-LlNLP7 | 5’-GAGCTCCACCGCGGTGGCGGCCGCTCTAGA  ATGTCGAACCCCGGCGCCGACGATT-3’ |
|  |  | 5’-GAATTCCTGCAGCCCGGGGGATCCCTCGAG TCAATCGACCGTGCTATCACATGAG-3’ |
| pGreenⅡ0800-LUC | ProLlHSFA3A-LUC | 5’-TACGACTCACTATAGGGCGAATTGGGTACC  CTCGAGGCATGTGCTCTGTATGTATATAAAACT-3’ |
|  |  | 5’-ATCAAGCTTATCGATACCGTCGACCTCGAG  CTCGAGGCATGTGCTCTGTATGTATATAAAACT-3’ |
| pGBKT7 | BD-LlNLP7 | 5’-GACCTGCATATGGCCATGGAGGCCGAATTC ATGTCGAACCCCGGCGCCGACGATT-3’ |
|  |  | 5’-GTTATGCGGCCGCTGCAGGTCGACGGATCC TCAATCGACCGTGCTATCACATGAG -3’ |
| pGBKT7 | BD- LlNLP7-P1 | 5’-GACCTGCATATGGCCATGGAGGCCGAATTC ATGTCGAACCCCGGCGCCGACGATT-3’ |
|  |  | 5’-GTTATGCGGCCGCTGCAGGTCGACGGATCC GAGGACGAAGGGCTGCCCGGAGGTG-3’ |
| pGBKT7 | BD- LlNLP7-P2 | 5’-GACCTGCATATGGCCATGGAGGCCGAATTC  ATGGGATCAGAAAATGATCTAATGG-3’ |
|  |  | 5’-GTTATGCGGCCGCTGCAGGTCGACGGATCC TCAATCGACCGTGCTATCACATGAG-3’ |
| pGADT7 | BD- LlNLP7-P3 | 5’-GACCTGCATATGGCCATGGAGGCCGAATTC ATGTCGAACCCCGGCGCCGACGATT-3’ |
|  |  | 5’-GTTATGCGGCCGCTGCAGGTCGACGGATCC  AAGGAGAGGCAAAGCTGAGAAGTCA-3’ |
| pGADT7 | BD- LlNLP7-P4 | 5’-GACCTGCATATGGCCATGGAGGCCGAATTC ATCAGTCTGGAGGTTCTCCAACAAT-3’ |
|  |  | 5’-GTTATGCGGCCGCTGCAGGTCGACGGATCC TCAATCGACCGTGCTATCACATGAG-3’ |
| pGADT7 | BD- LlNLP7-P5 | 5’-GACCTGCATATGGCCATGGAGGCCGAATTC ATGTCGAACCCCGGCGCCGACGATT-3’ |
|  |  | 5’-GTTATGCGGCCGCTGCAGGTCGACGGATCC GAGGACGAAGGGCTGCCCGGAGGTG-3’ |
| pGADT7 | BD- LlNLP7-P6 | 5’-GACCTGCATATGGCCATGGAGGCCGAATTC GATCCGCGCAGCAGCAAACTACTGC-3’ |
|  |  | 5’-GTTATGCGGCCGCTGCAGGTCGACGGATCC TTCAGCTAAAGCAGTTTGGCGCCCT-3’ |
| pGADT7 | BD- LlNLP7-P7 | 5’-GACCTGCATATGGCCATGGAGGCCGAATTC ATATTGGAGATCCTGACCATAGTCT-3’ |
|  |  | 5’-GTTATGCGGCCGCTGCAGGTCGACGGATCC ATTTCCAGATTGAGAACTTTGTAAG-3’ |
| pGADT7 | BD- LlNLP7-P8 | 5’-GACCTGCATATGGCCATGGAGGCCGAATTC GACGACTATATATTGGAGTTCTTCC-3’ |
|  |  | 5’-GTTATGCGGCCGCTGCAGGTCGACGGATCC  AAGGAGAGGCAAAGCTGAGAAGTCA-3’ |
| pGADT7 | BD- LlNLP7-P9 | 5’-GACCTGCATATGGCCATGGAGGCCGAATTC GATCCGCGCAGCAGCAAACTACTGC-3’ |
|  |  | 5’-GTTATGCGGCCGCTGCAGGTCGACGGATCC TCAATCGACCGTGCTATCACATGAG-3’ |
| pTRV2 | pTRV2-LlNLP7 | 5’-TGTGAGTAAGGTTACCGAATTCTCTAGA GGTTGTACTGGTTTGTGATGCAGAT-3’ |
|  |  | 5’-GGGCCTCGAGACGCGTGAGCTCGGTACC TGACTCGACTATCAATAAGACGAAC-3’ |
| pJG | pJG-LlNLP7 | 5’-GATGTGCCAGATTATGCCTCTCCCGAATTC ATGTCGAACCCCGGCGCCGACGATT-3’ |
|  |  | 5’-CTCTGGCGAAGAAGTCCAAAGCTTCTCGAG TCAATCGACCGTGCTATCACATGAG-3’ |
| pLacZi | pLacZi-LlHSFA3A | 5’-TGCTATCATTTCCTTTGATATTGGATCGGAATTC  CTCGAGGCATGTGCTCTGTATGTATATAAAACT -3’ |
|  |  | 5’-AGTTTTATATACATACAGAGCACATGCCTCGAG  CTCGAGGCATGTGCTCTGTATGTATATAAAACT-3’ |
| pEAQ | pEAQ-LlNLP7 | 5’-AGACAGTTGACTGTATCGCCGACCGGT ATGTCGAACCCCGGCGCCGACGATT-3’ |
|  |  | 5’-ATTTAATGAAACCAGAGTTAAAGGCCT TCAATCGACCGTGCTATCACATGAG-3’ |
| pEAQ | pEAQ-LlNLP7-P5 | 5’-AGACAGTTGACTGTATCGCCGACCGGT ATGTCGAACCCCGGCGCCGACGATT-3’ |
|  |  | 5’-ATTTAATGAAACCAGAGTTAAAGGCCT  GAGGACGAAGGGCTGCCCGGAGGTG-3’ |
| pEAQ | pEAQ-LlNLP7-P9 | 5’-AGACAGTTGACTGTATCGCCGACCGGT GATCCGCGCAGCAGCAAACTACT-3’ |
|  |  | 5’-ATTTAATGAAACCAGAGTTAAAGGCCT TCAATCGACCGTGCTATCACATGAG-3’ |

**Table S3** The promoter sequences used for EMSA assay.

| Promoter fragments | Sequence |
| --- | --- |
| LlNLP7-LlHSFA3A-bio-F | АССТСТТТGТТАТGССТТТТААСАССТТGССТАААGСТТАGGАTGAGGАT |
| LlNLP7-LlHSFA3A-bio-R | ATCCTCATCCTAAGCTTTAGGCAAGGTGTTAAAAGGCATAACAAAGAGCT |
| LlNLP7-LlHSFA3A-F | АССТСТТТGТТАТGССТТТТААСАССТТGCСТАААGСТТАGGATGAGGAT |
| LlNLP7-LlHSFA3A-R | АТССТСАТССТААGСТТТАGGCААGGTGTTAAAAGGCATAACAAAGAGGT |
| LlNLP7-mLlHSFA3A-bio-F | ACCTCTTTGTCGCAGTGGGGCCCACCTTGCCTAAAGCTTAGGGATGAGGAT |
|  |  |
| LlNLP7-mLlHSFA3A-bio-R | ATCCTCATCCTAAGCTTTAGGCAAGGTGGGCCCCACTGCGACAAAGAGGT |
|  |  |

**Table S4** The promoter sequences used for ChiP-qPCR assay.

| Name | Sequence |
| --- | --- |
| LlNLP7-C1 | 5’-GCATTATTGTCCACTCTCATTGCGA-3’ |
|  | 5’-CACGATTGGAGAAAGGTAACAT-3’ |
| LlNLP7-C2 | 5’-TGCCTACATTGGACTTAGGATC-3’ |
|  | 5’-ATCCTCATCCTAAGCTTTAGG-3’ |

**Table S5** Primers for DNA Identification.

| Gene name | Sequence |
| --- | --- |
| *LlNLP7* | 5’-CACCCAGTCCGCCCTGAGCAAAGACCCCA-3’ |
|  | 5’-TGCTTATATTTTCCAGCTTGAAGTGGAGC -3’ |
