# Supplementary material for: Lily NIN-like transcription factor LlNLP7 confers thermotolerance and promotes growth
Source: Mol Hortic. 2026 Aug 10;6:57. doi: 10.1186/s43897-026-00253-8 (PMC13455299; doi:10.1186/s43897-026-00253-8)
Supplement: Supplementary file 3 [file 43897_2026_253_MOESM3_ESM.docx]

**Materials and Methods**

**Plant materials and growth conditions**

In this study, the lily cultivar ‘White Heaven’ (*Lilium longiflorum* ‘White Heaven’) was used as the experimental material. The tissue-cultured lily seedlings were grown in a standard growth chamber at 22°C with a photoperiod of 16 h light and 8 h dark. Seeds of *Nicotiana benthamiana* were sown in MS medium, and after 10 days of germination, the seedlings were transplanted into seedling pots and cultivated in a greenhouse at 22/16°C with a 16-h light/8-h dark photoperiod.

**Stress treatments and gene expression analysis**

Uniformly sized and robust lily seedlings were selected for stress treatments and gene expression analysis. For heat stress treatment, the seedlings were incubated at 37°C for varying durations (0, 0.5, 1, 3, 6, 12 h) before leaf samples were collected. Total RNA was subsequently extracted from the collected leaves and reverse transcribed into cDNA using the HiScript II Kit (Vazyme, China). The expression levels of *LlNLP7* under different treatment conditions were quantified using real-time quantitative PCR (RT-qPCR), with the 18S rRNA of lily serving as the internal reference gene for RT-qPCR analysis (Table S1).

**Subcellular localization analysis**

The ORF (open reading frame) of *LlNLP7* was cloned into the pCAMBIA1300-GFP vector to produce a GFP fusion protein (Table S2). The empty vector pCAMBIA1300-C-GFP and the recombinant vector were individually introduced into the *Agrobacterium tumefaciens* strain GV3101. The RFP-NLS vector was used as a nucleus marker. Positive clones were screened and cultured, after which the resuspended bacterial solution (an infiltration buffer; 10 mM MgCl_2_, 200 μM acetosyringine, 10 mM MES, pH 5.6) was injected into *N. benthamiana* leaves. After 48 h of cultivation, leaf samples were collected and examined for GFP fluorescence signal localization using a laser scanning confocal microscope (LSM800, Zeiss, Germany).

**Transcriptional activity analysis**

The different fragments of *LlNLP7* were cloned into pGBKT7 to produce the BD fusion proteins. The recombinant plasmid was transformed into the yeast strain AH109 for transcriptional activity analysis, with yeast strains containing GAL4 and BD serving as positive and negative controls, respectively. The cultures were inverted at 30°C for 3 days, and positive clones were transferred to -Trp-His deficient medium for transcriptional activity assessment. Additionally, the *LlNLP7*, *LlNLP7*-P5, and *LlNLP7*-P9 were separately cloned into pEAQ to produce effector proteins. A reporter construct was generated by fusing 5×GAL4 UAS elements with a mini 35S promoter and cloning them into the vector pGreenII0800-LUC. The redesigned vectors were introduced into the *A. tumefaciens* strain GV3101 (psoup). Following resuspension of the bacterial solution and mixing in proportions, the mixture was injected into *N. benthamiana* leaves. Under normal growth conditions, after 48 h of cultivation, the injected leaves were collected, and the LUC signal was detected.

**Yeast one-hybrid assay**

Fragments of the *LlHSFA3A* promoter were individually cloned into pLacZi to construct the reporter vectors. The ORF of *LlNLP7* was also cloned into pJG to generate AD vectors. Subsequently, the corresponding vectors were co-transformed into the yeast strain Y187. The co-transformants were cultured on -Trp-Leu selective medium at 30°C for 3 days to screen for positive clones. Positive clones were then transferred to -Trp-Ura deficient SD medium to assess their ability to catalyze the substrate X-gal for color analysis.

**Electrophoretic mobility shift assay (EMSA)**

The ORF of *LlNLP7* was cloned into pGEX-4T-1 to produce a GST-tagged fusion protein. Expression of the fusion protein was induced in transformed *E. coli* BL21 cells (TIANGEN, China) by the addition of isopropyl β-D-1-thiogalactopyranoside (IPTG) (Sigma, USA). The accumulation of the tagged fusion protein was monitored using SDS-PAGE. The recombinant protein was purified using the GST protein purification kit (Thermo, USA). The EMSA probes were labeled with 5’-biotin (Table S3). The EMSA analysis was conducted using the Light Shift Chemiluminescent EMSA Kit (ThermoFisher, New York, USA).

**Dual-LUC reporter assay**

The ORF of *LlNLP7* was cloned into the vector pGreenII62-SK to construct the effector vector. The promoter of *LlHSFA3A* was cloned into pGreenII0800-LUC to create the reporter vector. Empty vectors pGreenII62-SK and pGreenII0800-LUC were used as negative controls. These vectors were transformed into *A. tumefaciens* GV3101 (pSoup). The mixed bacterial solution was infiltrated into the tobacco leaves for the determination of the LUC activity. Under normal growth conditions, the plants were cultivated for 60 h, after which the leaves were collected to detect LUC signals.

**Virus-induced gene silencing (VIGS)**

The coding region fragment of *LlNLP7* was amplified by PCR and cloned into pTRV2 to generate the pTRV2-LlNLP7 vector. Subsequently, pTRV1, pTRV2, and pTRV2-LlNLP7 were individually transformed into *A. tumefaciens* GV3101, and positive clones were screened and cultured. The cultures were then resuspended and mixed in appropriate ratios before being injected into the leaves of young lily seedlings. Under normal growth conditions, the plants were cultivated for 3 weeks, after which leaf tissues were collected for RNA extraction and RT-qPCR analysis to detect the gene silencing. The successfully silenced lily plants were selected and exposed to heat stress for the thermotolerance test. After heat stress, the leaves were harvested to determine the relative ion leakage and chlorophyll content.

**ChIP-qPCR assay**

Sterile lily bulbs were cultured on a callus-inducing medium consisting of MS supplemented with 2.0 mg·L^−1^ PIC (picloram), 0.2 mg·L^−1^ NAA (1-Naphthaleneacetic acid), 30 g·L^−1^ sucrose, and 7.0 g·L^−1^ agarose, adjusted to a pH of 5.8, to produce embryogenic callus. The *LlNLP7* ORF was cloned into the pCAMBIA1300-GFP vector to create a GFP-tagged fusion protein, with an empty GFP vector serving as a negative control. These constructs were transformed into *A. tumefaciens*, and bacterial solutions (OD600 = 1.0) were infiltrated into the lily calli under a vacuum. After vacuum release, the callus tissue was rinsed with sterile water and placed in a co-cultivation medium consisting of MS (NH_4_NO_3_-free) supplemented with 2.0 mg·L^−1^ PIC, 0.2 mg·L^−1^ NAA, 30 g·L^−1^ sucrose, 200 μM acetosyringone, 10 mM MES, and 7.0 g·L^−1^ agarose, adjusted to a pH of 5.8. This was incubated at 22°C in the dark for 3 days. Subsequently, 1.0 g of the infiltrated calli was collected and crosslinked in 1% (w/v) paraformaldehyde. The nuclei were extracted, and chromatin was fragmented into segments greater than 500 bp using sonication, followed by incubation and immunoprecipitation with a GFP antibody (Sigma, USA). The enrichment levels of the target DNA fragments were assessed by RT-qPCR using gene promoter-specific primers (Table S4).

**Stable transformation of *GFP*-*LlNLP7* in lily**

Lily embryogenic calli were utilized as the recipient material for transformation. The calli were submerged in a solution of *A. tumefaciens* for 10 min and then thoroughly dried by absorbing the excess bacterial solution from the calli’s surface using filter paper. Subsequently, the calli were transferred to a co-culture medium consisting of MS supplemented with 1.0 mg·L^−1^ PIC, 0.2 mg·L^−1^ NAA, and 100 μM acetosyringone. After co-cultivating in the dark at 25°C for 72 h, the infected calli were moved to a sterilized medium composed of MS supplemented with 1.0 mg·L^−1^ PIC, 0.2 mg·L^−1^ NAA, 400 mg·L^−1^ cefotaxime (Cef), and 30 mg·L^−1^ hygromycin (Hyg), and were continued to be cultured under dark conditions at 25°C. Two weeks later, the calli were transferred to a selection medium consisting of MS supplemented with 1.0 mg·L^−1^ PIC, 0.2 mg·L^−1^ NAA, and 30 mg·L^−1^ Hyg for cultivation. The calli were sub-cultured every two weeks, and any necrotic tissue was removed. After four weeks, somatic embryos began to emerge within the calli. The embryogenic culture containing these somatic embryos was then transferred to a somatic embryo germination medium consisting of MS with 0.5 mg·L^−1^ 6-benzylaminopurine (6-BA). Following four weeks of cultivation, the somatic embryos matured and developed into bulblets. These bulblets were subsequently transferred to MS medium for further cultivation, and transgenic plants were successfully obtained within two weeks. The positive transgenic plants were identified by GFP detection and RT-PCR assay (Table S5).

**Determination of Hydrogen Peroxide Content**

The content of hydrogen peroxide (H_2_O_2_) was determined using the Hydrogen Peroxide Assay Kit from (Nanjing Jiancheng Bioengineering Institute, China), with operations strictly following the manufacturer’s instructions. Three types of reaction tubes were set up: blank, standard, and sample tubes. The blank tube was supplemented with 0.1 mL of distilled water and 0.1 mL of 163 mmol/L H_2_O_2_ standard working solution, the standard tube with 0.1 mL of 163 mmol/L H_2_O_2_ standard working solution, and the sample tube with 0.1 mL of appropriately diluted test sample. Subsequently, 1.0 mL of Reagent 1 and 1.0 mL of Reagent 2 were added to each tube. After thorough vortex mixing, the absorbance (A) of each tube was measured at 405 nm using distilled water as the blank reference. Finally, the results were calculated according to the formula.

**Determination of Superoxide Anion Content**

The content of superoxide anion (O_2_^-^) was determined using the Superoxide Anion Content Assay Kit (Solarbio, China), with all procedures strictly following the manufacturer’s instructions. Three types of reaction tubes were prepared, namely the blank tube, standard tube, and sample tube: the blank tube was loaded with 0.5 mL of distilled water, the standard tube with 0.5 mL of standard solution, and the sample tube with 0.5 mL of appropriately diluted test sample. Subsequently, 0.4 mL of Reagent 1, 0.3 mL of Reagent 2, and 0.3 mL of Reagent 3 were added to each tube, respectively. After thorough vortex mixing, the tubes were incubated in a constant-temperature water bath at 37 ℃ for 20 min. Then, 0.5 mL of Reagent 4 (chloroform) was supplemented into each tube; the mixture was vortexed again and centrifuged at 8,000 rpm and 25 ℃ for 5 min. A total of 1 mL of the upper aqueous phase was carefully aspirated into a glass cuvette, and the absorbance value of each tube was measured at a wavelength of 530 nm with distilled water as the blank reference. Finally, the results were calculated according to the formula.
